# Genetic Analysis of Partially Resistant and Susceptible Chickpea Cultivars in Response to *Ascochyta rabiei* Infection

**DOI:** 10.3390/ijms25021360

**Published:** 2024-01-22

**Authors:** Amit A. Deokar, Mandeep Sagi, Bunyamin Tar’an

**Affiliations:** Crop Development Centre, Department of Plant Sciences, College of Agriculture and Bioresources, University of Saskatchewan, Saskatoon, SK S7N 5A8, Canada

**Keywords:** chickpea, ascochyta blight, resistance, RNA sequence, qPCR, candidate genes

## Abstract

The molecular mechanism involved in chickpea (*Cicer arietinum* L.) resistance to the necrotrophic fungal pathogen *Ascochyta rabiei* is not well documented. *A. rabiei* infection can cause severe damage in chickpea, resulting in significant economic losses. Understanding the resistance mechanism against ascochyta blight can help to define strategies to develop resistant cultivars. In this study, differentially expressed genes from two partially resistant cultivars (CDC Corinne and CDC Luna) and a susceptible cultivar (ICCV 96029) to ascochyta blight were identified in the early stages (24, 48 and 72 h) of *A. rabiei* infection using RNA-seq. Altogether, 3073 genes were differentially expressed in response to *A. rabiei* infection across different time points and cultivars. A larger number of differentially expressed genes (DEGs) were found in CDC Corinne and CDC Luna than in ICCV 96029. Various transcription factors including ERF, WRKY, bHLH and MYB were differentially expressed in response to *A. rabiei* infection. Genes involved in pathogen detection and immune signalings such as receptor-like kinases (RLKs), Leucine-Rich Repeat (LRR)-RLKs, and genes associated with the post-infection defence response were differentially expressed among the cultivars. GO functional enrichment and pathway analysis of the DEGs suggested that the biological processes such as metabolic process, response to stimulus and catalytic activity were overrepresented in both resistant and susceptible chickpea cultivars. The expression patterns of eight randomly selected genes revealed by RNA-seq were confirmed by quantitative PCR (qPCR) analysis. The results provide insights into the complex molecular mechanism of the chickpea defence in response to the *A. rabiei* infection.

## 1. Introduction

Chickpea (*Cicer arietinum L*.) is an important pulse crop grown on over a 14.8 M ha area with an annual production of 15.1 M tons in 2020 (FAOSTAT: http://www.fao.org/faostat/en/#data/QC; accessed on 14 April 2020). Chickpea is an important source of protein for resource-poor populations, especially in Asia and Africa. Chickpea production increased by 56% during the 2004–2013 period [1]; however, global productivity remained around one ton per hectare. Chickpea production is limited by several biotic and abiotic factors such as insect pests, diseases, drought and extreme temperatures; however, diseases alone have caused significant yield losses. Ascochyta blight is one of the most serious diseases of chickpea caused by the necrotrophic fungal *Ascochyta rabiei* (Pass.) Labr. Ascochyta blight occurrence has been reported in almost all chickpea growing areas around the world; however, in areas with cool and wet conditions, the disease can cause 100% yield loss [2].

The *A. rabiei* fungus infects all above-ground plant parts. The infection can occur on leaves, stems and pods. The disease can occur at any plant growth stage; however, the flowering-to-early-podding stage is the most vulnerable [2]. The *A. rabiei* spores germinate on leaflets and stems after 12–24 h of inoculation. Further, the germinated spores penetrate the plant surface through the leaf and stem cuticle as well as through stomatal openings. Eventually, the necrotic symptoms become visible, and mature pycnidia are observed within these brown necrotic lesions. The plant dies if the main stem is girdled at the collar region [3,4,5]. 

Ascochyta blight can be effectively managed by good agronomic practices and growing resistant cultivars. However, the development of chickpea cultivars with high levels of resistance to ascochyta blight has been limited largely due to a lack of source for strong resistance within the primary and secondary gene pools. Partial resistance to ascochyta blight has been identified and used successfully in different breeding programs to develop partially resistant cultivars. Partially resistant cultivars do not completely restrict the infection of *A. rabiei*; however, partial resistance delays the onset of the disease development and produces smaller and fewer lesions on leaves and stem compared to susceptible cultivars. Several factors affecting the expression of partial resistance have been identified including inoculum pressure and pathogenicity of isolates as well as plant age [6,7]. Genetic studies have identified several QTLs associated with ascochyta blight resistance [8,9,10,11,12], but these QTLs do not provide sufficient information to understand the resistance mechanism to ascochyta blight. Recent studies identified potential candidate genes ETHYLENE INSENSITIVE 3 and 4 (EIN3 and EIN4) within the proximity of the known QTL on chromosomes 2 and 4, respectively, which indicated the possible involvement of the genes within the ethylene metabolism in the resistance against ascochyta blight [13,14]. Additional candidate genes such as BED finger-NBS resistance protein, leucine-rich repeat domain protein and NBS TF, NBS-LRR, Receptor-like protein kinases, ethylene overproducing genes and BTB/POZ domain protein (BTB) were also identified within the resistance QTLs and may be involved in host resistance mechanisms [15,16,17]. 

RNA sequencing (RNA-Seq) has been widely used in profiling genome-wide gene expression in many plant species. RNA-seq can profile thousands of genes at once as well as identify novel transcripts and isoforms. Expression analysis using RNA-seq can provide a snapshot of actively expressed genes in response to specific conditions such as drought [18], high temperature [19], virus infection [20], bacterial disease [21] and fungal disease infection [22]. 

Only limited efforts have been pursued in chickpea to understand the molecular basis of ascochyta blight resistance using gene expression profiling. In the initial gene expression studies, the expression profiles of 20 defense-related genes have been analyzed in ascochyta-blight-resistant and -susceptible genotypes in response to *A. rabiei* [23]. Furthermore, 97 differentially expressed genes in response to *A. rabiei* in different resistant, moderately resistant, susceptible chickpea cultivars were identified using a cDNA microarray containing 756 probes [23]. Using PCR-based suppression subtractive hybridization (SSH) and macroarray strategy, 250 early responsive genes mainly involved in signaling and regulation of metabolic changes in response to *A. rabiei* infection were identified [24]. Recently, differential expressions of 15 defense-related genes in different resistant and susceptible genotypes were analyzed. Eight key genes were identified with differential expression profiles in resistant and susceptible genotypes [25]. Genome-wide identification of NBS-LRR genes and differential expression of NBS-LRR genes were also observed in response to ascochyta blight infections [26]. However, most of these studies covered only a limited number of known disease-associated candidate genes, and a global picture of gene expression profile is missing. The main objectives of this study were to examine the genome-wide expression profiles of two partially resistant chickpea cultivars (CDC Luna and CDC Corinne) and one susceptible cultivar (ICCV 96029) in response to *A. rabiei* infection in the early stages of infection at 24, 48 and 75 h post inoculation using the RNA-sequencing technique, and to identify potential candidate genes involved in the resistance mechanism.

## 2. Result and Discussion

### 2.1. Phenotypic Differences of Chickpea Cultivars in Response to Ascochyta Blight

Greenhouse screening with the local *A. rabiei* isolate AR170 showed that CDC Corinne and CDC Luna had an average disease score of 4.8 and 5.4, respectively, based on a 0–9 disease rating scale at 14 days after inoculation. The highly susceptible cultivar ICCV 96029 had a disease score of 8.8. The current disease scores are within the same range as the field scores of the three cultivars at different areas in western Canada. The current disease scores indicated that the greenhouse conditions and isolate used in the present study generated an effective condition for indoor ascochyta blight screening suitable for tissue sampling for genomic study.

The disease scores of CDC Corinne and CDC Luna ranging from 4.8 to 5.4 are generally classified as moderately resistant reaction. This reaction was characterized by 20–40% of its foliage area being affected by *A. rabiei*. The susceptible cultivar ICCV 96029 had more than 90% of its foliage area affected by the fungus. The results indicated that the partially resistant cultivars were able to significantly restrict the spread of *A. rabiei* compared to the susceptible cultivar. Molecular and cellular mechanisms of resistance mechanism to ascochyta blight in chickpea are still unknown. A delayed initial establishment of infection, and/or a delayed incubation period, i.e., from inoculation to the occurrence of the first symptoms, are some of the potential resistance mechanisms at the early stage that might be associated with partial resistance in chickpea against ascochyta blight. To analyze this possibility, we studied the transcriptomes from the two partially resistant and one highly susceptible chickpea cultivars in response to *A. rabiei* at an early stage of infection at 24, 48 and 72 hpi. 

### 2.2. Genome-Wide Transcriptome Sequencing

Tissue samples from the plants inoculated with *A. rabiei* isolate AR170 and plants from mock-inoculated control were collected at 24, 48 and 72 hpi. The samples at 24, 48 and 72 hpi were chosen because we were mainly interested in analyzing early-stage response of chickpea to *A. rabiei* infection in partially resistant and susceptible chickpea cultivars. The 24 hpi samples were chosen as a representative stage of maximum spore germination on the leaf surface, the 48 hpi as a representative stage of spore penetrations and the 72 hpi as a representative stage of necrotic lesions development in the mesophyll tissue [27]. Approximately 846 million Illumina raw reads were generated from the 12 RNA-seq libraries with an average of 71 million raw reads per library. More than 94% of the reads from the 11 libraries were with a high Q-score ≥ Q30, whereas one library (CDC Luna at 72 hpi) has low sequencing quality; therefore, was removed from further analysis (Table S1). On average, 1–3% of the cleaned reads were mapped to an *A. rabiei* genome, which was then removed from the subsequent analysis. Finally, 88 to 90% of the cleaned reads were mapped to the chickpea CDC Frontier V1.0 reference genome (Table S2). 

Overall, across all the RNA-seq libraries, we observed the expression of 62% (18,711) of the annotated genes, which varied from 65.5 to 69.4% in different libraries. On average, 34% of genes with the FPKM value of <0.1 were considered as very low- or no-expressed genes. A total of 7526 of these low-expressed genes were not detected in any of the libraries. These low-expressed genes may be tissue-specific and/or developmental-specific and, therefore, were not detected in this experiment. 

### 2.3. Differentially Expressed Genes in Response to A. rabiei 

Pairwise expression comparison between control (uninoculated samples) and *A. rabiei-inoculated* samples at different time points (24, 48 and 72 hpi) of each cultivar was conducted in order to identify differentially expressed genes per se in response to *A. rabiei* infection in resistant and susceptible chickpea cultivars. Overall, in all three cultivars and at three time points, a higher number of DEGs were identified in resistant cultivars CDC Corinne and CDC Luna than in the susceptible cultivar ICCV 96029. More specifically, 1051 genes were differentially expressed in ICCV 96026; whereas, 1132 and 2219 genes were differentially expressed in CDC Corinne and CDC Luna, respectively (Table 1 and Table S2). 

**Figure 1:**
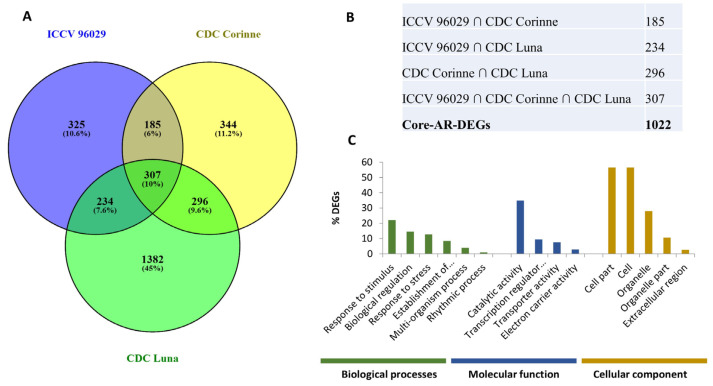
Differentially expressed genes in response to *Ascochyta rabiei* in ICCV 96029, CDC Corinne and CDC Luna. (**A**) Venn diagram showing the overlapping differentially expressed genes between ICCV 96029, CDC Corinne and CDC Luna cultivars. (**B**) A core set of DEGs were generated by grouping common DEGs between any two or all three cultivars as a set of genes differentially expressed in response to *A. rabiei.* (**C**) Gene Ontology (GO) enrichment analysis of core *Ascochyta rabiei*-responsive DEGs in three GO categories: biological processes, molecular function and cellular component.

After removing the redundant genes, a total of 3073 DEGs were identified as present at least at one time point and in any one cultivar. The 3073 DEG includes 1022 genes that can be considered as “core *A. rabiei*-responsive chickpea DEGs” (common between any two or all three cultivars) and 2051 genes as “cultivar-specific *A. rabiei*-responsive DEGs”. The cultivar-specific DEG included 325, 334 and 1382 genes exclusively identified in ICCV 96029, CDC Corinne and CDC Luna, respectively (Figure 1A,B). One hundred eighty-five DEGs were common in ICCV 96029 and CDC Corinne, whereas 234 DEGs were common in ICCV 96029 and CDC Luna. Two hundred ninety-six DEGs were common in CDC Corinne and CDC Luna (Figure 1A). At the later stage of infection, a higher number of genes were differentially expressed compared to the earlier time points. For example, in ICCV 96029 cultivar, a greater number of DEGs (534) were identified at 72 hpi, whereas 442 and 512 DEGs were identified at 24 and 48 hpi, respectively. In CDC Corinne, maximum numbers of DEGs (591) were identified at 72 hpi, whereas 566 and 364 DEGs were identified at 24 and 48 hpi, respectively. In CDC Luna, maximum numbers of DEGs (1952) were identified at 48 hpi followed by 1659 DEGs at 24 hpi (Table 1).

Subsequently, we performed Gene Ontology (GO) enrichment analysis of the DEGs to identify broad functional categories in response to *A. rabiei* infection. These DEGs were assigned to the biological processes, molecular function and cellular component according to the gene ontologies. The core *A. rabiei*-responsive DEGs were classified into 175 significant GO terms, of which 91, 34 and 50 GO terms belong to biological process, molecular function, and cellular component categories, respectively (Figure 1C, Table S3). In the biological process category, response to stimulus (GO:0050896), biological regulation (GO:0065007) and localization (GO:0051179) were significantly enriched. The GO term associated with a molecular function such as catalytic activity (GO:0003824), transcription regulator activity (GO:0030528) and transporter activity (GO:0005215) were significantly enriched. In the cellular component category, cell part (GO:0044464), cell (GO:0005623) and organelle (GO:0043226) were significantly enriched. The activation of genes associated with the GO in the biological process category of “response to stimuli” has been commonly observed under different biotic stresses [28,29]. Response to stimuli can be further expanded into response to stress, response to biotic and abiotic stimulus, response to chemical stimulus and response to external stimulus. The DEGs associated with the response to biotic stress include pathogenesis-related protein 1 (Ca_14762), thaumatin-like protein PR-5b (Ca_02420), L-myo inositol-1 phosphate synthase 1 (Ca_12683), nematode resistance protein (Ca_12499), hevein-like preproprotein-like (Ca_01237), and endochitinase A2-precursor (Ca_04687). Most of these proteins have antimicrobial activity and are induced in response to pathogen infection in different plants [30,31].

**Figure 2:**
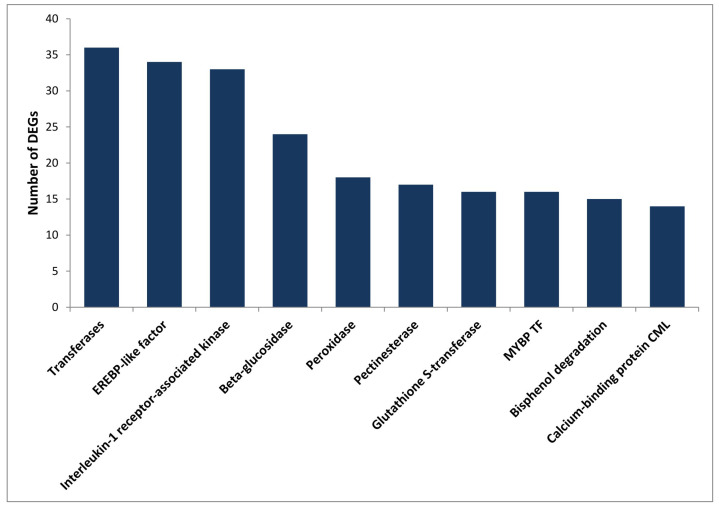
Top 10 of the KEGG Orthology (KO) annotations. Distribution of the top 10 KO terms from the chickpea DEGs in response to *A. rabiei* infection.

KEGG annotation of the DEGs was performed using the KAAS [32]. A total of 1978 (64.4%) DEGs were assigned with KO identifiers, and 131 pathways were associated with more than five KO identifiers. The major pathways included metabolic pathways (256), biosynthesis of secondary metabolites (153), plant hormone signal transduction (27), biosynthesis of amino acids (23), starch and sucrose metabolism (21) and plant–pathogen interaction (18) (Table S4). The DEGs were assigned with 914 KO terms, and the top 10 KO categories are presented in (Figure 2), while the complete list is presented in Table S4. These KO categories mainly included transferases (36), interleukin-1 receptor-associated kinase 4 (33), ERF (27), beta-glucosidase (24), peroxidase (18), pectinesterase (17) and glutathione S-transferase (16). Glucosidase, peroxidase, pectinesterase and glutathione S-transferase are classical cell wall proteins and their involvement in primary defence is well known in several plant fungal pathogen interactions [33]. The results of GO and KEGG annotations provide potential resources for further investigation of the process and pathway in response to *A. rabiei* infection in chickpea.

### 2.4. Differentially Expressed Genes in Partially Resistant Chickpea Cultivars 

To identify the DEGs specific to the partially resistant cultivars, we compared the DEGs in response to *A. rabiei* in ICCV 96029 (susceptible) with the two partially resistant cultivars (CDC Corinne and CDC Luna) separately. In total, 640 and 1678 DEGs were unequally identified in CDC Corinne and CDC Luna, respectively, compared to ICCV 96029 (Figure S1). Differential expression of these large numbers of genes between the resistant and susceptible cultivars in an early stage of infection (24 to 72 hpi) indicated rapid reprogramming of gene expression at the transcriptional level to regulate initial defense response against *A. rabiei.* A higher number of different DEGs in the two ascochyta-blight- resistant cultivars compared to the susceptible ICCV 96029 could possibly be associated with resistance mechanisms in CDC Corinne and CDC Luna against ascochyta blight. Additionally, 603 DEGs were commonly expressed in both resistant cultivars, whereas 529 and 1616 DEGs were uniquely identified in the resistant cultivars CDC Corinne and CDC Luna, respectively (Figure S1). Differential expression of a large number of diverse genes in both resistant cultivars also indicates the possibility of diverse transcriptional response in CDC Corinne and CDC Luna to *A. rabiei* infections. 

GO enrichment analysis was performed separately for the DEGs from ICCV 96029, CDC Corinne and CDC Luna in order to identify broad functional categories enriched in the partially resistant and susceptible cultivars under ascochyta blight infection (Figure S2). In the three different GO categories (molecular function, cellular component and biological process), 173, 176 and 144 GO terms were significantly enriched in ICCV 96029, CDC Corinne and CDC Luna, respectively. Among the enriched GO terms, 70 GO terms were common in all three cultivars, such as response to stimulus (GO:0050896), response to stress (GO:0006950), localization (GO:0051179), catalytic activity (GO:0003824), etc. Response to stimulus included a change in the state or activity of a cell such as enzyme production, secretion, and gene expression in reaction to fungal infection. Therefore, a higher number of DEGs were classified in this category across all three cultivars; however, in ICCV 96029, a slightly higher (24.5%) number of DEGs were identified in this GO class compared to CDC Luna and CDC Corinne (22.5 and 18.4%, respectively), indicating that the *A. rabiei* infection process initiated earlier in the susceptible cultivar than in the partially resistant cultivars. Seven GO terms, such as metabolic process (GO:0008152), regulation of biological quality (GO:0065008) and flavonoid biosynthetic process (GO:0009813), were uniquely enriched in the partially resistant cultivars CDC Corinne and CDC Luna. Genotype-specific GO enrichment has also been observed, such as 23 enriched GO terms that were only observed in CDC Luna including auxin transport (GO:0060918), hormone transport (GO:0009914) and the enzyme-linked receptor protein signaling pathway (GO:0007167). The GO term metabolic process (GO:0008152) was significantly enriched in partially resistant cultivars. This includes the pigment biosynthetic process, lipid metabolic process, phenylpropanoid metabolic process, etc. Many of these metabolic processes resulted in the production of structural and signaling molecules involved in plant defense [34]. Biosynthesis of cell wall components will enhance the physical barrier, hence preventing the pathogen from infecting the cell. This indicated that the modulation of metabolic processes significantly occurred in CDC Corinne and CDC Luna during the early stages of ascochyta blight infection. The observed differential expression response of different genes in susceptible and partially resistant cultivars and the process and pathways associated with these DEGs genes might be linked to the genotypic response (susceptibility and partial resistance) of the chickpea cultivars against *A. rabiei.* The observed differential gene response between the two partially resistant cultivars CDC Corinne and CDC Luna could be associated with the different resistance mechanisms in CDC Corinne and CDC Luna. Common QTL associated with ascochyta blight resistance on chromosome 3 in both CDC Corinne and CDC Luna as well as different QTL for ascochyta blight resistance on chromosome 5 in CDC Corinne and on chromosome 1 in CDC Luna were previously reported by Anbessa et al. [35]. This indicated the potential involvement of some shared as well as specific genes governing ascochyta blight resistance in CDC Corinne and CDC Luna. Our differential expression profiles also supported the possibility of different mechanisms of ascochyta blight resistance in CDC Corinne and CDC Luna.

### 2.5. Time-Dependent DEGs in Response to A. rabiei

**Figure 3:**
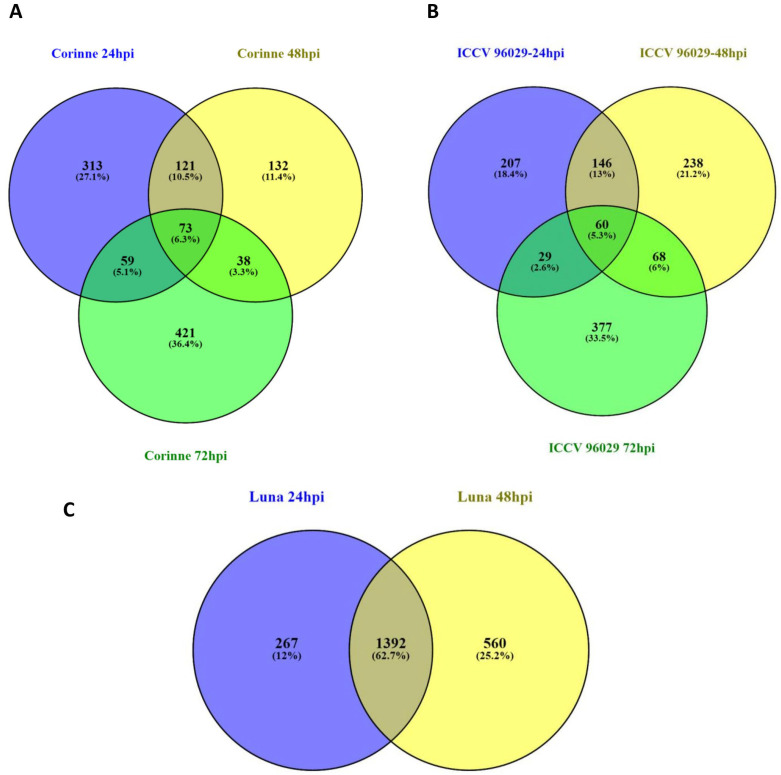
Comparative analysis of the changes in the chickpea transcriptomes in response to *A. rabiei* at three different time points after inoculation. Venn diagrams comparing differentially expressed genes between three different time points 24, 48 and 72 hpi in (**A**) ICCV 96029, (**B**) CDC Corinne and (**C**) CDC Luna.

To assess the differential transcriptome response of the resistant and susceptible cultivars at different time points after *A. rabiei* inoculation, the DEGs at 24, 48 and 72 hpi were compared using a Venn diagram (Figure 3). Comparisons of up- and down-regulated DEGs at 24, 48 and 72 hpi in ICCV 96029 showed that 56 up-regulated and 27 down-regulated DEGs had a similar pattern of expression in all three time points. However, the number of common DEGs between time points decreased with longer time after inoculation. For example, a higher number of common genes was observed between 24 and 48 (171 DEGS) than 48 and 72 (59 DEGs) hpi. The lowest was observed between 24 and 72 (28 DEGs), which indicated a rapid change in chickpea transcriptome in response to the increasing disease progress. Furthermore, 160 (98 up- and 62 down-regulated), 199 (141 up- and 58 down-regulated), 364 (237 up- and 127 down-regulated) DEGs were specifically identified at 24, 48 and 72 hpi, respectively (Figure 3A). In the partially resistant desi cultivar CDC Corinne, 39 and 38 DEGs were consistently up- and down-regulated, respectively. Whereas, 121 (51 up- and 70 down-regulated), 132 (94 up- and 38 down-regulated) and 421 (273 up- and 148 down-regulated) genes were specifically identified at the 24, 48 and 72 hpi time points, respectively, in the ICCV 96029 (Figure 3B). For the kabuli-type partially resistant cultivar CDC Luna, the DEGs at 24 and 48 hpi were compared. A total of 1362 (518 up- and 874 down-regulated) DEGs were consistently observed in these two time points. The cultivar-specific response, such as 267 (178 up- and 89 down-regulated) and 560 (194 up- and 336 down-regulated) DEGs, were identified specifically at 24 and 48 hpi, respectively (Figure 3C). The expressions of several *A. rabiei*-responsive genes in our experiment were mostly transient (time-dependent), with higher expression at some time points, and then returned to basal levels, and, therefore, undetected at other time points. The time-course expression analysis conducted in the present study demonstrated the benefit of the identification of diverse DEGs over the single time point expression analysis. Time-dependent differential expression of chickpea genes in response to *A. rabiei* has also been observed in another study [23]. 

### 2.6. Transcription Factors Regulation in Response to A. rabiei Infection

**Figure 4:**
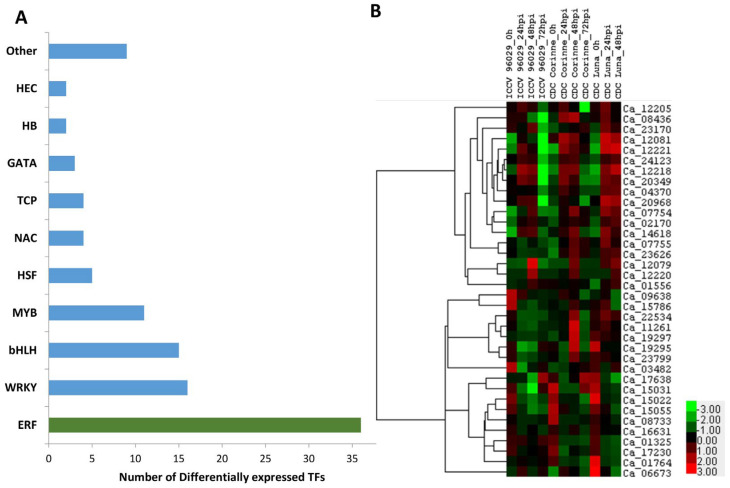
Differentially expressed chickpea transcription factors in response to *A. rabiei*. (**A**) Distribution of DEGs identified among TF gene families. (**B**) Heat map of significantly differentially expressed ERF TFs in response to *A. rabiei* infection in three chickpea cultivars (ICCV 96029, CDC Corinne and CDC Luna).

Transcription factors (TFs) are critical for the control of gene expression and their activity, which in turn determine how the cells function and respond to environmental stresses. Several classes of TFs play critical roles in response to a range of biotic and abiotic stresses. Out of 2235 known annotated transcription factors (TFs) in chickpea (PlantTFDB 4.0: http://planttfdb.cbi.pku.edu.cn/; accessed on 26 June 2020), 107 (4.8%) of the TFs were identified to be differentially expressed in response to *A. rabiei* in this study (Table S5). These TFs belong to 19 different transcription factor families, which mainly included 36 ethylene-responsive factors (ERF), 16 WRKY, 15 basic helix–loop–helix (bHLH), 11 MYB, 5 heat stress TFs (HSP), 4 NAC and others (Figure 4A).

ERF is one of the major stress-responsive TF families. In the present study, twenty-two ERFs were up-regulated in response to *A. rabiei* infection. Some of the ERF genes showed a differential expression pattern between the resistant and susceptible cultivars, such as the four ERF genes (Ca_20349, Ca_23170, Ca_24123 and Ca_08436) that were up-regulated in either CDC Corinne or CDC Luna but down-regulated in ICCV 96029 under *A. rabiei* infection. A few of the ERF genes (Ca_14618, Ca_12079, Ca_12220, Ca_12221 and Ca_12081) were up-regulated both in susceptible and either one of the resistant cultivars (Figure 4B). The chickpea genome contains more than 120 ERF TFs, which are further classified into ERF and DREB groups [36]. Several members of ERFs in different plants have been reported to be involved in diverse functions including regulation of developmental process, responses to biotic stress such as fungal pathogen infection and abiotic stress such as drought, salt and cold [37]. Overexpression and transcriptional activation studies suggested that the ERF genes are broadly involved in biotic stress responses, whereas the DREB genes are broadly involved in abiotic stress responses. Plant defense responses initiate the activation of one or more signaling pathways such as salicylic acid (SA), jasmonic acid (JA), abscisic acid (ABA) and ethylene (ET). The JA and ET are predominantly involved in resistance to necrotrophic pathogens. Several members of ERF are responsive to both JA and ET pathways and work as a point of integration of JA and ET pathways [38,39]. Overexpression of many ERF genes such as ERF1, AtERF4, AtERF5, AtERF6, ERF59 (ORA59) and ERF96 in *Arabidopsis* confers resistance to necrotrophic fungal pathogen [38,39,40,41]. Overexpression of homologs of different ERF genes from different plant species also confirms the role of ERFs in necrotrophic disease resistance, such as the overexpression of the B3-type Medicago MeERF1-1 showing enhanced resistance against the necrotrophic root-infecting pathogens *Rhizoctonia solani* and *Phoma medicaginis* [42]. Another member of the pathogen-induced B3-type ERF TF of wheat (TaERF1) conferred enhanced resistance to the necrotrophic fungus *Rhizoctonia cerealis* [43]. The members of ERF TFs can regulate gene expression either as a repressor or activator of downstream defense genes and showed either an up- or down-regulation pattern under stress conditions [38]. These results indicated that the ERF regulated pathway is one of the major defense-related pathways involved in the necrotrophic disease resistance in *Arabidopsis* and other plant species. Differential expression of 36 different ERFs in the initial phase of *A. rabiei* infection indicated that the ERFs and ethylene pathway may play an important role in the ascochyta blight response in chickpea.

WRKY is another plant-specific TF family, which is largely involved in the transcriptional regulation during plant immune responses [44]. In our expression analysis, 16 WRKY genes were differentially expressed in response to *A. rabiei*. Two chickpea WRKY genes (Ca_00420 and Ca_24738) were up-regulated in both resistant and susceptible cultivars, whereas eight WRKY genes (Ca_15224, Ca_13524, Ca_08975, Ca_04141, Ca_08614, Ca_06984, Ca_24738 and Ca_05782) were up-regulated in either of the two resistant cultivars. Ca_13524, which was up-regulated in the resistant cultivar CDC Luna, is a homolog of *Arabidopsis* WRKY33 TF that functions as a positive regulator of disease resistance against two necrotrophic fungi, *Alternaria brassicicola* and *Botrytis cinerea* [45]. *Arabidopsis* WRKY33 regulates the biosynthesis of camalexin, which is a secondary metabolite with antimicrobial activity. Its accumulation has been reported to play defensive functions against several necrotrophic pathogens [46]. Differential expression of the majority of the WRKY genes (49 out of 79 WRKY genes) in response to *Pseudomonas syringae* infection in *Arabidopsis* has also been observed [47]. WRKY domains specifically bind with W-boxes (TTGACC/T), a pathogen response element found in several defense-related genes such as pathogenesis-related (PR) genes and, thereby, regulate expression of defense-associated genes. Several of these PR genes were also differentially expressed in our experiment. Apart from the *Arabidopsis*, the involvement of WRKY genes in immune response in other plant species has also been reported such as the rice WRKY gene OsWRKY4 being strongly induced in response to the infection of rice necrotrophic fungus *Rhizoctonia solani* and exogenous application of JA and ET. Overexpression of the same gene induced resistance against the *Rhizoctonia solani* through the JA/ET-dependent signaling pathway [48]. Expression of the grape WRKY gene (VvGRKY2) was also associated with enhanced resistance against multiple necrotrophic fungal pathogens [49]. 

Ten MYB TFs were differentially expressed under *A. rabiei* infection in this study. Of them, five MYB genes (Ca_03498, Ca_13024, Ca_02593, Ca_16710 and Ca_17627) were up-regulated in response to *A. rabiei.* Ca_03498 was strongly up-regulated in ICCV 96029 but showed down-regulation in CDC Luna. Significant induction of *Arabidopsis* (AtMYB108) and wheat MYB gene (TaRIM1) in the response to necrotrophic pathogen has been observed [50,51]. Overexpression of these genes also showed increased resistance in transgenic *Arabidopsis* and wheat against *Botrytis cinerea* and *Rhizoctonia cerealis,* respectively. 

Four NAC genes (Ca_08257, Ca_16379, Ca_18090 and Ca_05227) were up-regulated in response to *A. rabiei.* The Ca_05227 gene, which is a homolog of the *Arabidopsis* ANAC072 gene, showed up-regulation in CDC Luna. Differential expression of several other *Arabidopsis* NAC genes (NAC002, ANAC019, ANAC055, ANAC081 and ANAC092) in response to necrotrophic pathogen has been reported [52]. 

Apart from these major TFs, several other TFs (Table S5) were also differentially expressed in response to *A. rabiei,* which eventually regulated the expression of several other downstream pathogenesis-related genes to induce disease resistance or susceptibility. 

### 2.7. Pathogen Recognition

#### 2.7.1. Differentially Expressed Receptor-like Protein Kinases (RLKs)

Receptor-like protein kinases (RLKs) are transmembrane receptors proteins that play a key role in the innate immune system by regulating recognition and early responses to diverse plant pathogens [53]. The RLKs are pattern-recognition receptors (PRRs) that detect pathogen-associated molecular patterns (PAMPs) and, upon binding of their related elicitors, initiate PAMP-triggered immunity (PTI). Chickpea contains more than 600 RLKs [54]. In our experiment 75 putative RLKs were differentially expressed under *A. rabiei* infection (Table S6). Out of the differentially expressed 75 RLKs, 10, 17 and 60 RLKs were detected in ICCV 96029, CDC Corinne and CDC Luna, respectively. Two RLK (Ca_13431, Ca_14621) and Ca_18810 were up- and down-regulated in all three cultivars and at all time points. Some of the RLKs such as Ca_23302, Ca_10945, Ca_12145 and Ca_09104 were up-regulated specifically in either of the two resistant cultivars CDC Corinne and CDC Luna. A few RLKs such as Ca_15777, Ca_23304, Ca_12960, and Ca_08031 were significantly detected only in susceptible cultivar ICCV 96029. Another RLK gene encoding cysteine-rich receptor-like protein kinase (Ca_05531) was up-regulated at 24 and 48 hpi in both resistant and susceptible cultivars; however, the fold change was higher (4.1) in CDC Luna than in ICCV 96029 (2.5). The cell-wall-associated receptor kinase (Ca_10945) was also significantly induced in response to *A. rabiei* infection in both CDC Corinne and CDC Luna. Along with these RLKs, 18 other protein kinases were also differentially expressed in response to *A. rabiei,* including CBL-interacting protein kinases, serine/threonine-protein kinases, calcium-dependent protein kinases and cyclin-dependent kinases. One of the CBL-interacting protein kinases (Ca_12145) was up-regulated by 3.5 and 4-fold in CDC Corinne and CDC Luna, respectively.

Several members of LecRKs in *Arabidopsis*, tobacco and tomato have been reported to be involved in resistance to Phytophthora pathogens. Overexpression of the *Arabidopsis* LecRK gene *LecRK-VI.2* in *Arabidopsis* and tobacco also showed enhanced resistance to the necrotrophic pathogens [55,56]. The studies of LecRK genes and their homologs in *Arabidopsis* showed the key role of LecRK genes in broad-spectrum disease resistance in plants. Induction of these genes in our experiments also suggested that the chickpea LecRKs might be involved in the stress signal transduction process during the ascochyta blight infection in chickpea. The majority of the information about RLKs was derived from the biotrophic pathogen interaction, such as the rice RLK genes *Xa21* that encoded RLK- LRR protein and conferred resistance to the bacterial blight pathogen *Xanthomonas oryzae* pv *oryzae* [57]. The *Arabidopsis* flagellin-sensitive 2 (FLS2) and BRI1-associated kinase 1 (BAK1) both encode RLK-LRR and form a heterodimer in the presence of flg22 and contribute to resistance against bacterial pathogens [58]. Other studies have enlightened the involvement of PRR perception of PAMPs in the plant resistance against necrotrophic pathogens. The *Arabidopsis* AtCERK1 and AtLYK4 encoding a lysin motif receptor-like kinase (LysM-RLK) recognized chitin [59,60]; AtRLP42 encoding a leucine-rich repeat receptor-like protein recognized fungal endopolygalacturonases (PGs) [61]; RPL30 *Arabidopsis* receptor-like protein 30 recognized sclerotinia culture filtrate elicitor1 (SCFE1) [62]; and the LeEIX1 and LeEIX2 genes encoding tomato LRR-RLP recognized fungal protein ethylene-inducing xylanase [63] and mediated PTI to necrotrophic fungus. These findings and differentially expressed RLKs in our experiment indicated the potential involvement of RLKs in the early stage of ascochyta blight infection in chickpea.

#### 2.7.2. Calcium-Binding Protein (CML)

CML-binding proteins have been identified to be involved in the initial signaling events associated with plant defense responses. In the present study, 13 differentially expressed CML genes were identified in response to *A. rabiei.* The majority of the differentially expressed CML genes, except for Ca_19593, Ca_02142 and Ca_06443, were down-regulated after *A. rabiei* inoculation. Two CML genes (Ca_19593 and Ca_06443) were up-regulated in either one of the resistant cultivars. Another CML gene, Ca_02142, was also up-regulated in both ICCV 96029 and CDC Corinne at 72 hpi; however, the fold change was higher in resistant cultivar CDC Corinne (Table S7). The multiple isoforms of CaM/CML genes, especially Ca_03226, Ca_08764, Ca_10354 and Ca_22380, were markedly down-regulated in the susceptible genotype ICCV 96029. This down-regulation of CaM/CML isoforms genes was not observed in either resistance genotypes CDC Luna or CDC Corinne. Rapid induction of cytoplasmic free calcium (Ca^2+^) levels and thereby activation of defense responses is a common response to pathogen infection in plants [64]. Expression of several Calmodulin (CaM), CMLs and CaM-binding protein in response to early pathogen infection have been reported in many plants [65]. The silencing of the expression of tobacco CaM gene NtCaM13 enhanced plant susceptibility to viral, bacterial and fungal pathogens [66]. On the contrary, overexpression of the pepper CaM1 gene activated the expression of defense-related genes in pepper leaves, leading to local resistance to pathogens [67]. All these observations indicated the involvement of CaM/CML isoforms in multiple aspects of plant immunity. Similarly, the observed differential expression of the multiple chickpea CaM genes in the early phase of *A. rabiei* infection in our study suggested the involvement of chickpea CaM genes in ascochyta blight reaction.

### 2.8. Pathogenesis-Related Proteins

#### 2.8.1. Peroxidase

Peroxidase activity was strongly induced during the course of plant–pathogen interactions and played an important role in several defense-related processes such as hypersensitive reaction, lignification, protein cross-linking and phytoalexin production [68,69]. Changes in peroxidase activity during the early phase of necrotrophic pathogen infection has been observed in several plant species including chickpea [68,70,71]. In the present study, 16 peroxidase genes were identified as differentially expressed in response to *A. rabiei* infection (Table S8). Nine peroxidase genes were only detected in CDC Corinne and CDC Luna, whereas the remaining genes were detected in all three cultivars at some time points. Eight peroxidase genes (Ca_04365, Ca_15641, Ca_07982, Ca_07983, Ca_04125, Ca_03373, Ca_16223 and Ca_08963) were up-regulated in response to *A. rabiei* infection. Differential expression patterns between cultivars were also observed for peroxidase 73-like gene Ca_09339, which was down-regulated in ICCV 96029 and up-regulated in CDC Corinne and CDC Luna in response to *A. rabiei* infection. Differential expressions of peroxidase gene during pathogen response have also been observed in many plant species [72,73]. Overexpression of some of the peroxidase genes has been shown to enhance disease resistance, confirming the role of peroxidase genes in host–pathogen interaction [72,73]. For example, overexpression of rice peroxidase gene *OsPrx114* improved disease resistance against necrotrophic pathogen through the elevated levels of PR genes, rapid removal of H_2_O_2_ during the oxidative burst and lignin formation [73]. Overall, the involvement of peroxidase in disease resistance against necrotrophic pathogen has been mainly correlated with the role of peroxidase in cell wall strengthening through the deposition of lignin, suberin, callose and extension, which helps in improving resistance to wall-degrading enzymes produced by necrotrophic pathogens and provides a mechanical barrier to toxin entrance and physical penetration of the pathogen. 

#### 2.8.2. Glutathione S-Transferase

Glutathione S-transferase (GSTs) plays an important role in plant disease resistance [74]. We identified 13 differentially expressed GSTs in our experiment (Table S8). Seven GSTs were up-regulated in *A. rabiei*-infected samples, and two of them (Ca_21197 and Ca_0573) were only detected in resistant genotype CDC Corinne and CDC Luna. Involvement of the antioxidative activity of GSTs in reducing the cellular damage caused by pathogens or cell death caused by free radicals produced during the hypersensitive response has been mainly associated with the involvement of GSTs in disease resistance [74]. In chickpea, GSTs has been shown to be involved in detoxifying Solanapyrone A and B produced *by A. rabiei* [75]. Up-regulation of GST genes in response to *Ascochyta lathyri* infection in grass pea [76] and *Didymella pinodes* in field pea [77] reflected the important role of GSTs in disease resistance.

#### 2.8.3. Chitinase

The involvement of chitinase in plant disease resistance was based on direct involvement of chitinase in the degrading fungal cell wall or by releasing compounds that activate PTI. In the present study, eight putative chitinase genes were up-regulated in *A. rabiei*-infected samples. Ca_01237 is a heven-like pathogenesis-related protein 4 (PR4) with antifungal chitin-binding activity that was up-regulated in all three cultivars in response to *A. rabiei* infection (Table S8). Three other chitinase genes (Ca_09920, Ca_05018 and Ca_04405) were up-regulated only in ascochyta-blight-resistant cultivar CDC Luna. Three chitinase genes (Ca_02709, Ca_14789 and Ca_02708) were down-regulated in response to *A. rabiei* infection. The induction of chitinase activity in leaves and pods of both resistant and susceptible chickpea cultivars after being inoculated with *A. rabiei* has been observed earlier [30]. Transgenic plants overexpressing chitinases genes alone or in combination with other PR genes showed enhanced resistance against several necrotrophic pathogens [31]. Higher expression of the PR-3 and PR-4 genes in the *Arabidopsis* plants overexpressing ERF96 genes showed enhanced resistance to necrotrophic pathogens such as the fungus *Botrytis cinerea* and the bacterium *Pectobacterium carotovorum* [78]. This indicated that ERF-mediated gene regulation of PR genes improved disease resistance in *Arabidopsis*. The differential regulation of different chitinase genes in response to *A. rabiei* indicated the possible involvement of these genes in ascochyta blight resistance in chickpea. Down-regulation of chitinase genes in the *A. rabiei*-infected plants may be associated with the mechanism to overcome the plant defense response and consequently facilitated the progression of the *A. rabiei* infection, which we detected in both resistant and susceptible cultivars.

### 2.9. Phenylpropanoid Pathway

Enhanced activation of the phenylpropanoid pathway in host plants infected with different types of pathogen has been observed in many plant species [34]. Differential expression of the key enzyme in this pathway including phenylalanine ammonia-lyase (PAL), chalcone synthase (CHS), isoflavone reductase (IFR) and Flavanone 3-Hydroxylase (F3H) in resistant and susceptible chickpea cultivars under *A. rabiei* infection has been reported in chickpea [79]. Phenylalanine ammonia-lyase catalyzes the first step in the phenylpropanoid pathway and is an important regulatory point between primary and secondary metabolism associated with disease resistance [34]. In the present study, three PAL genes (Ca_17144, Ca_08478 and Ca_09817) were up-regulated in the *A. rabiei*-infected samples at 72 hpi in both ICCV 96029 and CDC Corinne (Table S9). Five isoflavone reductase genes (Ca_20342, Ca_20578, Ca_04776, Ca_02018 and Ca_02019) were up-regulated in all three cultivars after *A. rabiei* infection. Up-regulation of two Flavanone 3-Hydroxylase genes (Ca_20295, and Ca_12950) was also observed in resistant cultivar CDC Corinne. Differential expression of IFR genes in chickpea under *A. rabiei* and *Fusarium oxysporum* has been also observed in previous studies [80]. Induced PAL, IFR and F3H activities in both resistant and susceptible cultivars have been observed in many studies including chickpea [79,81].

Chalcone synthase (CHS) gene expression is often induced in plants under the different types of environmental and developmental stimuli, including fungal pathogen infection which caused accumulation of different flavonoid and isoflavonoid compounds with antimicrobial, insecticidal and antioxidant activity [82]. In the present study, five chalcone synthase genes (Ca_11408, Ca_08294, Ca_11821, Ca_11822 and Ca_08546) were differentially expressed under the *A. rabiei* infection (Table S9). Two chickpea CHS genes (Ca_11821 and Ca_11822) were up-regulated in all three cultivars; however, the fold change in CDC Corinne and CDC Luna was higher than in the susceptible cultivar ICCV 96029. Similar induction of CHS genes in the cell suspension culture of both resistant (ILC 3279) and susceptible (ILC 1929) chickpea cultivars after 4 h of induction with *A. rabiei* elicitor has been observed; however, a twofold-higher gene expression level has been detected in the cells of resistant cultivar ILC 3279 than in the cells of susceptible cultivar ILC 1929 [80]. Differential expression of the CHS gene in response to *A. rabiei* race3 has been also observed in ascochyta-resistant (Kc 218848) and -susceptible (Kc 217854) chickpea cultivars. Early induction of the CHS gene at 3 hpi and then at 24 and 168 hpi has been observed in resistant cultivar Kc 218848; however, the expression of CHS in susceptible cultivar has not been detected significantly [79]. Overall differential expression of key genes in phenylpropanoid pathway in resistant and susceptible chickpea cultivars observed in our experiment and previous studies indicated the involvement of CHS in ascochyta blight resistance.

### 2.10. Cytochrome P450 (CYP)

P450s are involved in a variety of metabolic pathways and induced by several environmental stresses. In the present study, 23 cytochrome P450 (CYP) genes were differentially expressed in response to *A. rabiei* infection (Table S10). Nineteen CYP genes were detected only in CDC Corinne and CDC Luna, whereas the remaining six were detected in all three cultivars (Table S10). The majority of the CYP genes showed up-regulation in response to *A. rabiei.* The cytochrome P450 86A2 gene (Ca_05757) showed the cultivar-specific expression pattern, i.e., up-regulated in ICCV 96029 but down-regulated in CDC Corinne and CDC Luna. Up-regulation of two cytochrome p450 genes in response to *Botrytis cinerea* in *Solanum lycopersicoides* at 24 and 48 h after inoculation has been observed [83]. The soybean CYP82 gene family (GmCYP82A3) was strongly induced in response to *Phytophthora sojae* infection and methyl jasmonate (MeJA) or ethephon (ETH) applications. Transgenic tobacco plant overexpressing GmCYP82A3 exhibited strong resistance to *Botrytis cinerea* and *Phytophthora parasitica* and less sensitivity to JA, indicating the possible involvement of GmCYP82A3 in disease resistance via JA and Ethylene Signaling Pathway [84]. In barley, CYP96B22 gene has been shown to be involved in disease resistance through the regulation of penetration resistance of barley plants to host and non-host isolates of *Magnaporthe oryzae* [85]. Differential regulation of chickpea CYPs in response to *A. rabiei* provides a sign of involvement of CYPs in ascochyta blight defense response.

### 2.11. RT-qPCR Validation of Differentially Expressed Genes in Response to A. rabiei Infection

**Figure 5:**
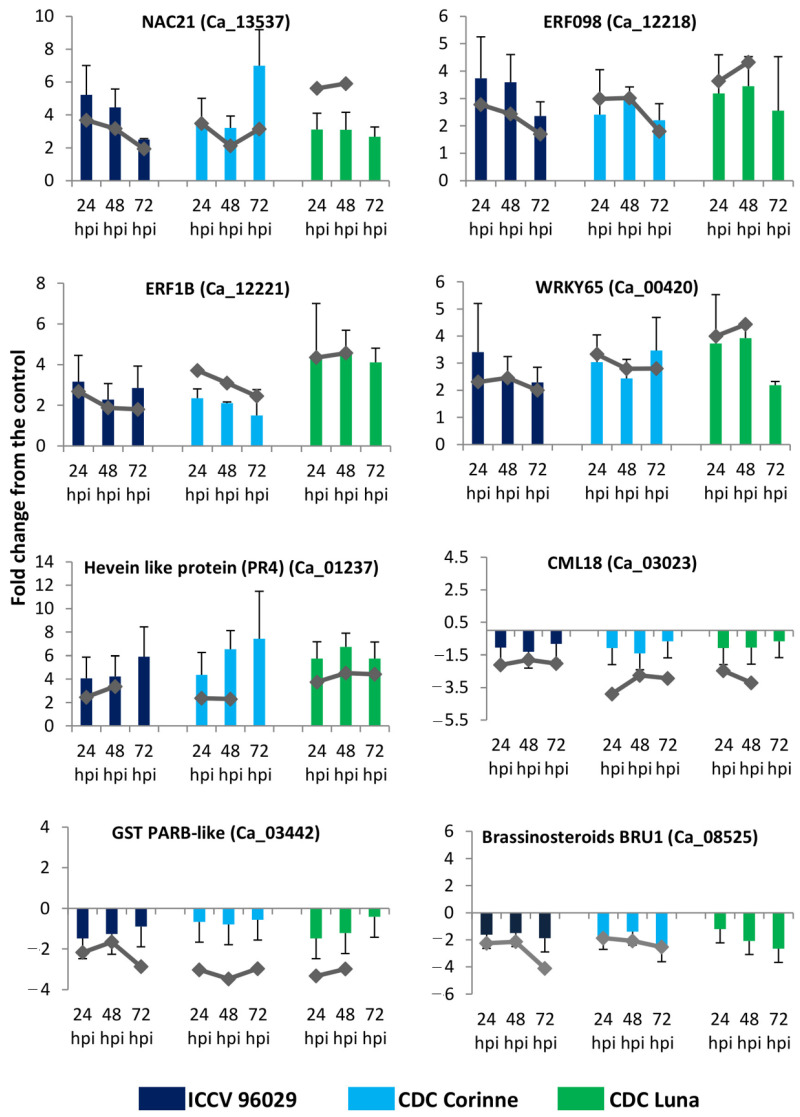
RNA-seq and RT-qPCR fold change values. Combined bar and line plots showing a comparison of RT-qPCR and RNA-seq fold change values for eight randomly selected differentially expressed genes.

To confirm the RNA-seq results for differential gene expression, the expression patterns of a set of selected genes were assessed using RT-qPCR. The GAPDH was used to normalize the relative quantities of the target genes across the different time points and genotypes. This reference gene has also previously been found to be the most stable housekeeping gene for chickpea–*A. rabiei* interaction [26]. Overall, in the qPCR analysis, we observed general similarities and a clear pattern of differential expression (up- and down-regulation), as observed in the RNA-seq analysis (Figure 5). Genes that were up-regulated in response to *A. rabiei* infection such as ethylene-responsive transcription factor ERF098 (Ca_12218), WRKY transcription factor 65 (Ca_00420) in RNA-seq analysis were also found up-regulated in the qPCR analysis. Similarly, the genes that were down-regulated such as brassinosteroid-regulated protein BRU1 (Ca_08525) also demonstrated to be down-regulated in response to *A. rabiei* infection. There were slight differences in the fold change values, which could be due to the obvious differences in the qPCR and RNA-seq methods in terms of data collection, quantification and fold calculation. 

## 3. Materials and Methods

### 3.1. Plant Materials

Two partially resistant chickpea cultivars (CDC Luna and CDC Corinne) and one susceptible cultivar ICCV 96029 were used to identify differential transcriptomes in response to ascochyta blight infection. CDC Luna is a kabuli-type cultivar partially resistant to ascochyta blight with an average disease rating score of 4.6 on a 0–9 scale [86]. CDC Corinne is a desi-type cultivar partially resistant to ascochyta blight with an average disease rating score of 3.9 on a 0–9 scale [87]. Both cultivars were developed and released by the Crop Development Centre (CDC), University of Saskatchewan, Canada, for cultivation in western Canada. ICCV 96029 is a desi-type chickpea cultivar highly susceptible to ascochyta blight. This cultivar was developed at the International Crops Research Institute for the Semi-Arid Tropics (ICRISAT) at Hyderabad, India, and it was released as a super-early-maturing chickpea cultivar for the Asian region. ICCV 96029 had an average ascochyta blight disease score of 8.5 on a 0–9 scale under western Canadian growing conditions. In the 0–9 scale, 0 represents no disease and 9 represents plants dead [88]. The disease scores of 0–3 represent resistant, scores 4–6 are considered moderately resistant, and scores 7–9 are susceptible [10]. 

### 3.2. A. rabiei Inoculations and Sample Collection for RNA-Seq

A moderately aggressive *A. rabiei* isolate AR170 collected from Saskatchewan, Canada, was used to inoculate the chickpea plants. Conidial suspension of AR170 was prepared as described by Armstrong-Cho et al. [89]. Briefly, 10-day-old AR170 cultures grown on oatmeal agar and 100 mg chloramphenicol were used to prepare the conidial suspensions by flooding cultures with sterile deionized water to a final concentration of 2 × 10^5^ conidia mL^−1^, as determined with hemocytometer. 

Fifteen-day-old seedlings (8–10 internode stage) of the three cultivars were inoculated with approximately 3 mL of AR170 conidial suspension culture or mock-inoculated with water (as control) using an airbrush sprayer. Inoculated plants were covered with transparent polyethylene covers and kept in a high-humidity chamber for 24 h and then were transferred to a greenhouse with an overhead mist irrigation facility. Tissues from AR170 and mock-inoculated plant samples were collected in three biological replications at 24, 48 and 72 h post inoculations (hpi). Plant samples were immediately frozen in liquid nitrogen and stored at −80 °C. Additional inoculated plants from the same inoculation experiment were kept in the greenhouse mist bench for two weeks and scored for the disease response. These plants were used to confirm the success of the inoculation procedure.

### 3.3. RNA-Seq

Total RNA was isolated using hybrid TRIzol (Invitrogen) RNA extraction and the RNeasy (Qiagen) column purification method. The quality of the RNA was checked with a NanoDrop^®^ (Thermo Scientific. Waltham, MA, USA) and Agilent 2100 bioanalyzer (Agilent Technologies. Santa Clara, CA, USA). The RNA samples with OD260/OD280:1.8-2.0 and RNA integrity numbers ≥ 7 were used for library construction. RNA-Seq libraries were prepared from total RNA using poly(A) enrichment and then fragmented and primed for cDNA synthesis. The first strand was created using reverse transcriptase and random primers, and the second strand was then synthesized to generate double-stranded cDNA. After generating double-stranded cDNA, terminal repair and ligation of poly(A)/sequencing oligonucleotide adaptors were carried out. The second-strand cDNA was excised by UNG enzyme. Fragments with the expected size were purified and then amplified by PCR. The purified PCR products were sequenced with the Illumina HiSeq™ 2500 at Funomics Global Inc. (www.funomics.com; Saskatoon, SK, Canada).

### 3.4. RNA-Seq Data Analysis

Raw reads were processed to remove low-quality reads (Q-score < Q30), Ns reads, adapter contaminants and rRNA sequences. Reads that passed the quality filter parameters were then mapped to the *A. rabiei* transcriptome and reference genome AscRab 1.0 [90]. Reads mapped to the ascochyta genome were removed, and the remaining reads were used for further analysis. Filtered reads were mapped to the chickpea reference genome (CDC Frontier V1.0). The TopHat and Cufflinks Version 2.2.0 [91] package were used to calculate gene expression levels with default settings. Differentially expressed genes (DEGs) were selected using the NOIseq method, which is a non-parametric approach for the identification of differentially expressed genes from normalized count data [92].

### 3.5. Downstream Data Analysis

*Arabidopsis* homologs of chickpea DEGs were identified using the BlastP program with an E-value of 1 × 10^−3^ Gene Ids and were then submitted to AgriGO online toolkits [93] for gene ontology (GO) term enrichment analysis using the Singular Enrichment Analysis (SEA) module. Fisher’s exact test was applied for the enrichment analysis with the false discovery rate (FDR) of 0.05 to identify the significant functional GO terms.

KEGG annotation was performed using the single-directional blast-hit (SBH) method in KAAS web-server [32]. The obtained KEGG Orthology (KO) identifiers or K numbers were used to reconstruct the KEGG pathways maps using KEGG web-server (http://www.genome.jp/kegg/ko.html; accessed on 1 March 2020). 

### 3.6. Validation of RNA-Seq Analysis by qPCR

qPCR assays were performed to confirm the RNA-seq results. One microgram of total RNA from each sample (the same samples as for RNA-seq) was used to synthesize cDNA using a SensiFAST cDNA synthesis kit according to the manufacturer’s instruction (Bioline, Inc. Cincinnati, OH, USA). The SensiFAST SYBR No-ROX kit was used for qPCR using a BIO-RAD CFX384 real-time PCR detection system (Bio-Rad laboratories), in accordance with the manufacturer’s protocols. The data were collected from three biological and three technical replications. Initially, we tested 5 reference genes including *18SrRNA*, Elongation factor [*Ef1α*], *GAPDH*, Initiation factor [*IF4a*] and *ACTIN*. *GAPDH* was selected and used as a reference gene to normalize the relative quantities of the target genes because of its consistency across different time points and genotypes. The comparative CT method was used for quantification of gene expression, and fold change was calculated using 2^−∆∆CT^ [94]. The list of the selected genes and primers used for validation are listed in Table S11.

## 4. Conclusions

The results of the current study provide new insight into the transcriptomic response of chickpea to *A. rabiei* infection. The overall DEGs among the partially resistant and susceptible chickpea cultivars to *A. rabiei* infection are summarized in Figure 1, which includes genes involved in initial pathogen recognition, signal transduction, transcription regulations and pathogenesis-related proteins. A higher number of genes were differentially expressed at the later stage (72 hpi) than at the earlier time points. A higher number of DEGs were also found in the two ascochyta blight moderately resistant cultivars (CDC Corinne and CDC Luna) compared to the susceptible cultivar (ICCV96029), which could possibly be associated with resistance mechanisms against ascochyta blight in these cultivars. The information of genes and pathways associated with disease resistance in the moderately resistant chickpea cultivars will serve as the potential target for selection to accelerate breeding for resistance and minimize the losses caused by *A. rabiei.*

## Figures and Tables

**Table 1:** Summary of *A. rabiei*-responsive differentially expressed genes (DEGs) in three chickpea cultivars ICCV 96029 (susceptible), CDC Corinne and CDC Luna (partially resistant).

Time Interval	*A. rabiei*-Responsive DEGs in ICCV 96029			*A. rabiei*-Responsive DEGs in CDC Corinne			*A. rabiei*-Responsive DEGs in CDC Luna		
	Up-Regulated	Down-Regulated	Sub-Total	Up-Regulated	Down-Regulated	Sub-Total	Up-Regulated	Down-Regulated	Sub-Total
0–24 hpi	267	175	442	201	365	566	696	963	1659
0–48 hpi	343	169	512	211	153	364	712	1240	1952
0–72 hpi	348	186	534	373	218	591	-	-	-
	Non-redundant DEGs in ICCV 96029		1051	Non-redundant DEGs in CDC Corinne		1132	Non-redundant DEGs in CDC Luna		2219
	Non-redundant DEGs in response to *A. rabiei* in all three cultivars								3073

## Data Availability

The raw data supporting the conclusions of this article will be made available by the authors on request.

## Supplementary Materials

The following supporting information can be downloaded at: https://www.mdpi.com/article/10.3390/ijms25021360/s1.
