# Supplementary figures and images for: Genetic Analysis of Partially Resistant and Susceptible Chickpea Cultivars in Response to Ascochyta rabiei Infection
Source: Int J Mol Sci. 2024 Jan 22;25(2):1360. doi: 10.3390/ijms25021360 (PMC10816841; doi:10.3390/ijms25021360)

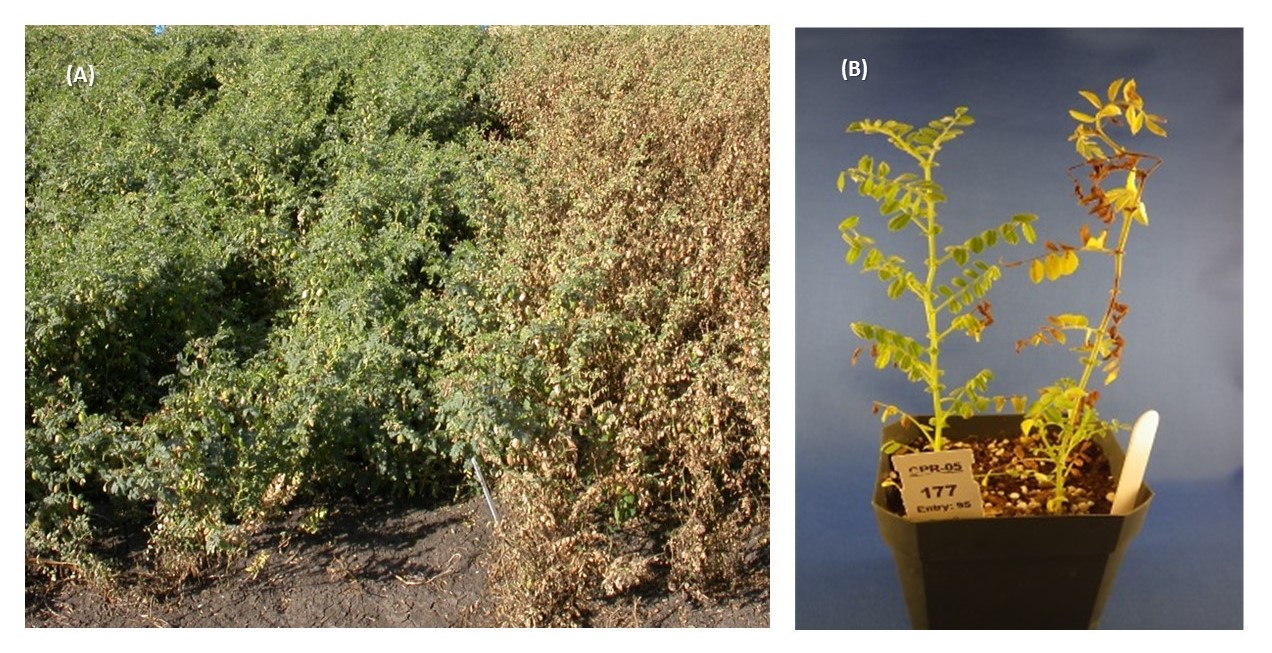

Supplement: Supplementary file 1 [file ijms-25-01360-s001.zip › Updated reordered Supplementary Tables (ijms-2766409-proof 2)/Figure S1..jpg]

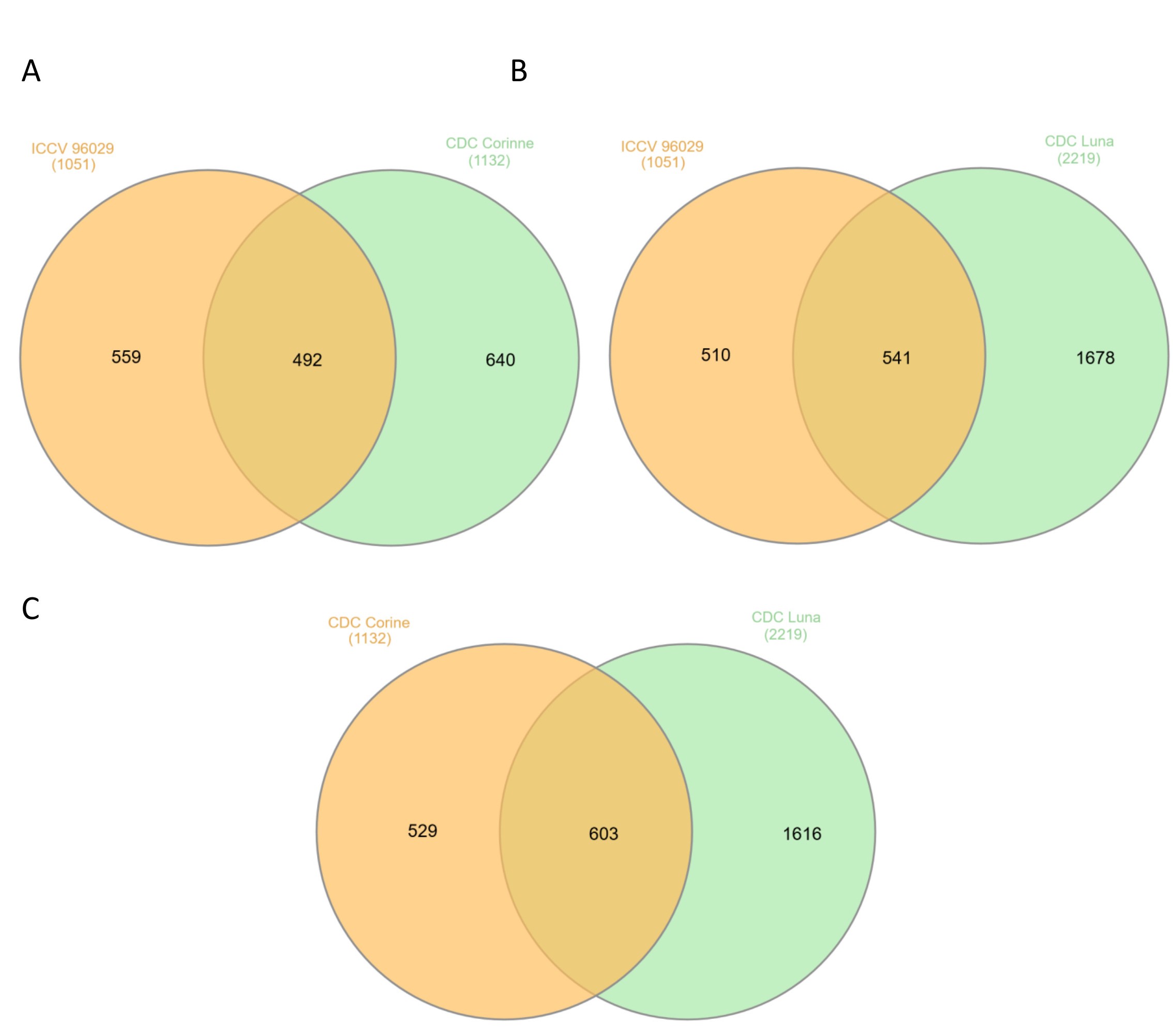

Supplement: Supplementary file 1 [file ijms-25-01360-s001.zip › Updated reordered Supplementary Tables (ijms-2766409-proof 2)/Figure S2.jpg]

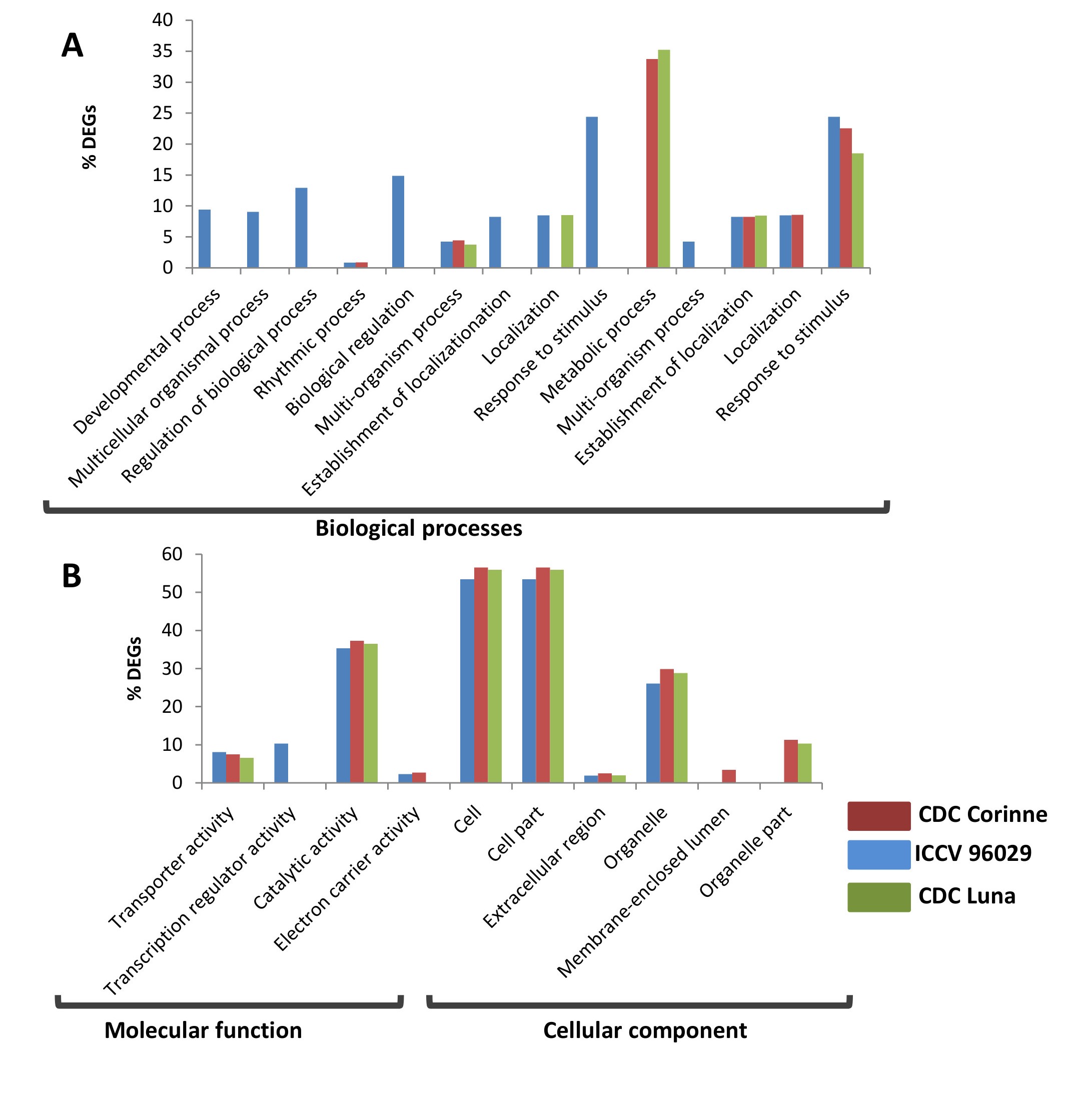

Supplement: Supplementary file 1 [file ijms-25-01360-s001.zip › Updated reordered Supplementary Tables (ijms-2766409-proof 2)/Figure S3.jpg]
